# Protective effect of manganese in cadmium-induced hepatic oxidative damage, changes in cadmium distribution and trace elements level in mice

**DOI:** 10.2478/v10102-010-0013-3

**Published:** 2010-06

**Authors:** Vladislav Eybl, Dana Kotyzová

**Affiliations:** Department of Pharmacology and Toxicology, Charles University in Prague, Faculty of Medicine in Pilsen, CZ-301 00 Pilsen, Czech Republic

**Keywords:** cadmium, oxidative stress, manganese, essential elements

## Abstract

Oxidative tissue damage is considered an early sign of cadmium (Cd) toxicity and has been linked with carcinogenesis. Manganese(II)-at low doses, was found to act as a potent antioxidant against oxidative stress in different in vitro systems producing lipid peroxidation conditions. The present study investigates in vivo antioxidant effects of Mn^2+^ pretreatment in acute Cd intoxication with regard to lipid peroxidation, antioxidant defense system and cadmium distribution in the tissues of mice. Four groups of male mice (n=7–8) were used: Cd group was injected sc a single dose of CdCl_2_ · 2½ H_2_O · (7 mg/kg b.w.); Cd+Mn group was treated *ip* with MnCl_2_ · 4H_2_O (20 mg/kg b.w.) 24 hours before Cd intoxication; Mn group received manganese treatment only; Control group received saline only. Twenty-four hours after Cd intoxication an increased lipid peroxidation (*p*<0.05), depleted GSH level (*p*<0.01), increased activity of GSH-Px (*p*<0.05) and inhibited CAT activity (*p*<0.01) were found in Cd-treated group compared to controls. Manganese(II) pre-treatment either completely prevented (LP, GSH, GSH-Px) or significantly attenuated (CAT) these changes. Manganese(II) treatment alone decreased LP, enhanced hepatic GSH level and had no effect on antioxidant enzymes compared to control group. A significant increase of Cd concentration in the liver and decreased Cd concentration in the kidneys and testes were found in Cd+Mn treated mice compared to Cd-only treated group. The effect of manganese may result from a different metallothionein induction in particular organs. Manganese(II) pretreatment attenuated the interference of cadmium with Ca homeostasis, the alteration in Zn and Cu levels remained mostly unaffected.

## Introduction

Cadmium (Cd) represents a carcinogenic metal (IARC, 1993) and it is a serious environmental and industrial pollutant. Industrial emissions, fertilization and cigarette smoking represent important sources of cadmium exposure for humans. In the body cadmium accumulates predominantly in the liver, kidneys, reproductive tissues, etc. (WHO, 1992). The adverse effects of cadmium include oxidative damage in tissues. This effect is considered as an early sign of its toxicity and has been linked with carcinogenesis (Walkes, 2000; Valko *et al*., 2006).

Manganese (Mn) is an essential element required in living organisms both as an activator and a constituent of several enzymes. Manganese is an important cofactor of mitochondrial superoxide dismutase, an antioxidant enzyme which scavenges oxygen free radicals (Macmillan-Crow & Cruthirds, 2001). At higher doses, manganese causes neurodegenerative disorders, which are attributed to oxidative stress (Dobson *et al*., 2004; HaMai & Bondy, 2004; Santamaria & Sulsky, 2010). However, at low doses manganese, differently from transition metals such as iron and copper, was found to act as a potent antioxidant in the protection against oxidative stress (Sziraki *et al*., 1995). In vitro experiments revealed the ability of Mn(II) to scavenge oxygen free radicals generated in differently mediated lipid peroxidation conditions (Cavallini *et al*., 1983; Coassin *et al*.,^1992^; Sziráki *et al*., 1999; Hussain & Ali, 1999; Valachová *et al*., 2010).

Pretreatment with low doses of Mn(II) has been found to produce tolerance to Cd-induced lethality and hepatotoxicity in mice and rats (Goering & Klaassen, 1985; Kotyzova *et al*., 1990). The experiments on rat liver mitochondria incubated with cadmium indicated that Mn^2+^ ions may protect from Cd-induced lipid peroxidation and the inhibition of antioxidant enzymes (Casalino *et al*., 2002a, b; Pillai & Gupta, 2005).

The aim of the present study was to investigate the in vivo effect of low-dose manganese(II) pretreatment on acute Cd-intoxication with regards to lipid peroxidation, antioxidant defense system and cadmium distribution in the tissues of mice.

## Materials and methods

### Animals and treatment

Male CD mice weighing 21–22g were purchased from Anlab (Prague, CZ). The animals were housed in a temperature and humidity controlled room with 12h light/dark cycles and free access to standard pellet diet and drinking water. After 5 days of adaptation, the animals were assigned to four groups of 7–8 animals: I. Control group; II. Cd group; III. Cd+Mn group; IV. Mn group. The mice in Cd group were injected *sc* cadmium chloride (CdCl_2_ · 2½H_2_O, Lachema Brno, CZ) at a single dose of 7 mg/kg of body weight. The animals from Cd+Mn group received a single dose of manganese chloride (MnCl_2_ · 4H_2_O, Lachema Brno, CZ), at a dose of 20 mg/kg body weight, administered *ip* 24 hours prior to cadmium intoxication. The mice in Mn group received manganese treatment only; the control group received saline only. Twenty-four hours after the treatment with Cd the mice were killed by decapitation in ether anesthesia. The liver, kidneys and testes were excised, rinsed in ice-cold saline and used immediately or stored frozen at –70°C until analyzed.

The experimental treatment protocol was approved by the local Animal Care and Use Committee. The investigation conforms to the Guide for the Care and Use of Laboratory Animals published by the U.S. National Institute of Health.

### Analytical procedures

Lipid peroxidation (LP) was estimated in liver homogenates (10% w/v) by measuring the malondialdehyde (MDA) production formed in the thiobarbituric acid reaction (Mihara & Uchiyama, 1978). Reduced glutathione (GSH) level was estimated in the deproteinized supernatant fraction of liver homogenate using 5,5′-dithiobis(2-nitrobenzoic acid) and reading absorption at 412 nm (Sedlak and Lindsay, 1968). Glutathione peroxidase (GSH-Px) activity was assayed in liver homogenates by a coupled test system, in which glutathione reductase is employed for regeneration of GSH and butylhydroperoxide used as the acceptor substrate. The decrease in NADPH concentration was registered photometrically at 340 nm (Günzler *et al*., 1974). Catalase (CAT) activity was estimated by following the decomposition of H_2_O_2_ directly by the decrease in extinction of hydrogen peroxide at 240 nm (Aebi *et al*., 1972).

For cadmium and essential element estimation the tissues were dry-ashed in a muffle furnace under temperature-controlled program overnight; the ash was solubilized with 3M HCl. Appropriately diluted samples were analyzed for Cd, Zn, Cu, Fe, Mn, Ca and Mg by flame- or grafite furnace technique of atomic absorption spectrometry (SpectrAA 220 FS Varian, Australia Ltd.). Analytical accuracy was monitored by assaying reference samples of NBS reference material 1577 Bovine Liver. The element contents are expressed in µg/g of wet tissue weight.

The data are expressed as means ± SD. Significant differences between experimental groups were estimated using unpaired Student's *t*-test. *p-*values <0.05 were considered statistically significant.

## Results

**Figure 1:**
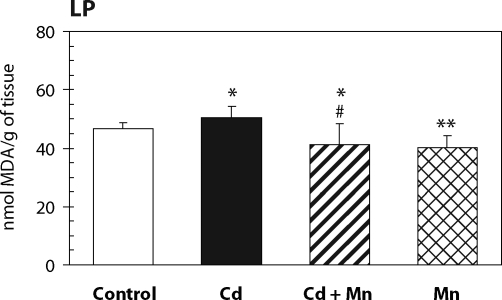
The effect of Mn^2+^ on the lipid peroxidation in the liver of Cd-intoxicated mice. Data represent mean ± SD; *N*=7 animals in control and Mn groups, *N*=8 animals in Cd and Cd+Mn groups; Significant differences: *p<0.05 vs. control group, #*p*<0.05 vs. Cd group.

**Figure 2:**
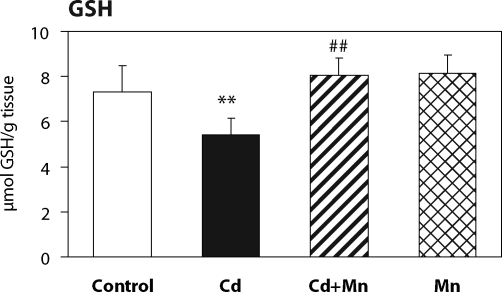
The effect of Mn^2+^ on glutathione depletion in the liver of Cd-intoxicated mice. Data represent mean ± SD; *N*=7 animals in control and Mn groups, *N*=8 animals in Cd and Cd+Mn groups; Significant differences: **p<0.01 vs. control group, ##*p*<0.01 vs. Cd group.

**Figure 3:**
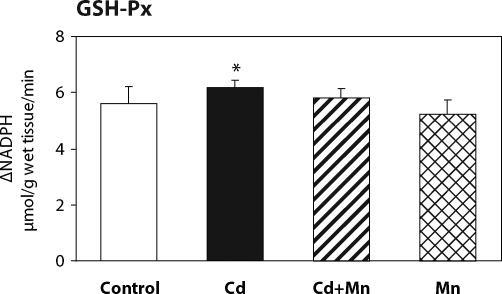
The effect of Mn^2+^ on GSH-Px activity in the liver of Cd-intoxicated mice. Data represent mean ± SD; *N*=7 animals in control and Mn groups, *N*=8 animals in Cd and Cd+Mn groups; Significant differences: *p<0.05 vs. control group.

**Figure 4:**
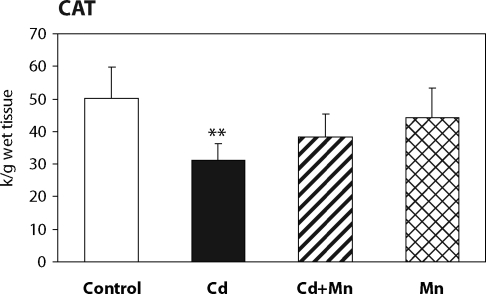
The effect of Mn^2+^ on catalase activity in the liver of Cd-intoxicated mice. Data represent mean ± SD; *N*=7 animals in control and Mn groups, *N*=8 animals in Cd and Cd+Mn groups; Significant differences: **p<0.01 vs. control group.

The effect of Mn^2+^ pretreatment on hepatic lipid peroxidation in Cd-intoxicated and control mice is presented in Figure 1. Twenty –four hours after cadmium administration a significant increase in hepatic lipid peroxidation was measured. When mice were pretreated with Mn^2+^ the increase in lipid peroxidation was prevented. The MDA production in Mn^2+^ treated animals was lower than in controls. As shown in Figure 2, the pretreatment with Mn^2+^ also completely prevented the decrease of hepatic GSH level caused by cadmium. The effects of Mn^2+^ on the activity of antioxidant enzymes – glutathione peroxidase and catalase – are shown in Figure 3 and Figure 4. Pretreatment with Mn^2+^ prevented the effect of cadmium on GSH-Px activity and significantly attenuated the inhibition of CAT activity caused by Cd-intoxication. Manganese treatment alone had no effect on the activity of antioxidant enzymes when compared to control group.

The data in Table 1 demonstrate the accumulation of cadmium in the tissues of Cd-treated mice, 24h after the single administration of cadmium chloride. A significant increase of Cd concentration in the liver, and decreased Cd concentration in the kidneys and testes were found in Cd+Mn treated mice compared to Cd-only treated group. The effect of manganese treatment on zinc, copper, iron, manganese, calcium and magnesium concentration in the tissues of control and Cd-intoxicated mice is shown in Table 2. In Cd treated mice the pretreatment with manganese significantly decreased Ca level in the liver and the testes, and Zn level in the kidneys, enhanced due to Cd intoxication. Other alteration, i.e. the increase of Zn level in the liver and the decrease of Cu, Zn, and Mg level in the testes, remained unaffected by Mn^2+^ pretreatment. Manganese treatment alone decreased Cu and Mg level in the liver and Cu and Zn level in the kidneys.

**Table 1:** The effect of manganese on cadmium distribution in the tissues of Cd-intoxicated mice.

	Liver	Kidneys	Testes
**Control**	≤0.2	≤0.2	≤0.02
**Cd**	20.2 ± 2.6	9.2 ± 1.5	0.42 ± 0.07
**Cd+Mn**	25.1 ± 3.4 ^##^	6.0 ± 0.6^##^	0.23 ± 0.04^##^

Values in µg/g of tissue wet weight; presented as means ± SD of *N*=7 animals in control group, *N*=8 animals in Cd and Cd+Mn groups;

Significant differences: ^##^ *p*<0.01 *versus* Cd-group

**Table 2:** The effect of manganese treatment on trace element concentration in the tissues of control and Cd-intoxicated mice.

	Ca	Mg	Zn	Cu	Fe	Mn
**LIVER**						
**Control**	30.5 ± 1.8	246 ± 21	22.5 ± 3.1	3.94 ± 0.42	81 ± 15	0.89 ± 0.12
**Cd**	45.0 ± 9.5^**^	235 ± 29	33.8 ± 4.9^**^	4.25 ± 0.49	105 ± 32	0.92 ± 0.11
**Cd+Mn**	35.9 ± 4.5^*^^#^	243 ± 32	34.9 ± 4.1^**^	4.25 ± 0.56	90 ± 19	1.41 ± 0.27^**^
**Mn**	30.5 ± 1.4	216 ± 8^*^	21.7 ± 1.8	3.40 ± 0.28^*^	71 ± 13	1.08 ± 0.10^*^

**KIDNEYS**						
**Control**	52.6 ± 5.8	211 ± 10	16.8 ± 0.8	3.27 ± 0.19	35.6 ± 2.3	1.38 ± 0.13
**Cd**	53.5 ± 6.5	217 ± 10	19.4 ± 0.6^**^	3.44 ± 0.24	35.6 ± 4.0	1.20 ± 0.11^*^
**Cd+Mn**	50.4 ± 2.4	204 ± 4	17.6 ± 0.3^#^	3.37 ± 0.25	35.2 ± 3.2	3.10 ± 0.44^**^
**Mn**	55.6 ± 9.8	210 ± 5	15.7 ± 0.6^*^	3.07 ± 0.21^*^	34.5 ± 2.2	3.10 ± 0.43^**^

**TESTES**						
**Control**	41.8 ± 5.9	165 ± 6	19.4 ± 2.9	1.23 ± 0.10	11.6 ± 0.8	n.a.
**Cd**	181 ± 32^**^	64 ± 11^**^	14.5 ± 2.4^**^	0.96 ± 0.12^**^	39.2 ± 19^**^	n.a.
**Cd+Mn**	139 ± 8^**^^##^	65 ± 6^**^	13.6 ± 0.8^**^	0.98 ± 0.05^**^	24.7 ± 2.6^*^	n.a.
**Mn**	39.4 ± 2.8	160 ± 4	17.3 ± 0.6	1.17 ± 0.05	11.8 ± 1.4	n.a.

Values in mg/g of tissue wet weight; presented as mean ± SD; *N*=7 animals in control and Mn groups, *N*=8 animals in Cd and Cd+Mn groups;

Significant differences: *p<0.05 and

**p<0.01 *versus* control group;

^#^ *p*<0.05 and

^##^ *p*<0.01 *versus* Cd-group;

n.a.=not analysed

## Discussion

There is evidence that acute Cd exposure is associated with hepatic oxidative stress. Oxidative stress is characterized by increased lipid peroxidation and/or altered nonenzymatic and enzymatic antioxidant system. The present study was designed to investigate the effect of manganese(II) pretreatment on Cd-induced oxidative liver injury.

Manganese is an essential element present in several enzymes, some of which are involved in redox processes. Dual behavior of Mn(II) depends on the quantity of manganese administered. High doses cause oxidative injury, while at low concentration Mn^2+^ ions effectively scavenge superoxide and hydroxyl radicals (Shiraki *et al*., 1995, Hussain & Ali, 1999; Santamaria & Sulsky, 2010).

In the present study, a protective effect of manganese(II) in Cd-induced oxidative liver damage was demonstrated in an experiment in vivo. The dose of MnCl_2_ administered in our experiment was previously shown to decrease lipid peroxidation in the liver of rats when administered *ip* for 30 days (Chen *et al*., 2004). The Cd-induced liver oxidative damage was characterized by an increase in hepatic lipid peroxidation accompanied by a depletion of the main endogenous antioxidant molecule – glutathione, and by the alterations in the activity of antioxidant enzymes – glutathione peroxidase and catalase. The antioxidant effect of Mn^2+^ pretreatment completely prevented or significantly attenuated the adverse effects of cadmium on lipid peroxidation and antioxidant defense system in the liver of Cd-intoxicated mice. Manganese(II) administration alone decreased the production of thiobarbituric acid reactive substances and exerted no effect on hepatic GSH level and on the activities of antioxidant enzymes – glutathione peroxidase and catalase.

As regards the effect of manganese on Cd distribution, few investigations have been referred. The results from our study show that MnCl_2_ administration prior to Cd intoxication significantly increased Cd accumulation in the liver with concomitant decrease of Cd level in the kidneys and the testes. This effect of Mn(II) might result from a different metallothionein induction in particular organs. This is in accordance with suggestions of Goerring & Klaassen (1985) that Mn^2+^ pretreatment alters the hepatic subcellular distribution of cadmium with more Cd^2+^ bound to metallothionein in the cytosol. Accordingly, also the hepatic level of manganese was enhanced in Cd+Mn treated mice compared to Mn-only treated group. Recent studies of Himeno *et al*. (2002; 2009) utilizing kinetic and competition analyses revealed that Cd and Mn share a common pathway for entering the cells and that the transport system for Mn is utilized for cellular Cd uptake in mammals.

As regards the effect of Mn^2+^ pretreatment on Cd-induced changes in essential elements levels, our data show that Mn^2+^ pretreatment significantly attenuated the interference of cadmium with Ca homeostasis in the liver and the testes and abolished the effect of cadmium on Zn level in the kidneys. Only moderate changes were found in the essential element balance in Mn-only treated mice compared to control animals.

In conclusion, the results from this study demonstrate the antioxidant effect of manganese(II) in Cd-induced hepatic oxidative injury in mice. Further investigation on the relation between Mn accumulation and resistence to oxidative stress and on the factors influencing Mn/Cd transport in mouse cell are needed to elucidate the molecular basis of this protective effect.

## References

[CIT0001] Aebi H, Bergmeyer HU (1972). Catalase. Methods of Enzymatic Analysis.

[CIT0002] Casalino E, Calzaretti G, Sblano C, Landriscina C (2002a). Molecular inhibitory mechanisms of antioxidant enzymes in rat liver and kidney by cadmium. Toxicology.

[CIT0003] Casalino E, Valzaretti G, Sblano C, Landriscina V, Felice Tecce M, Landriscina C (2002b). Antioxidant effect of hydroxytyrosol (DPE) and Mn2+ in liver of cadmium-intoxicated rats. Comp Biochem Physiol Part C.

[CIT0004] Cavallini L, Valente M, Bindoli A (1983). Inhibition of lipid peroxidation by manganese. Inorg Chim Acta.

[CIT0005] Chen MT, Sheu JY, Lin TH (2004). Protective effects of manganese against lipid peroxidation. J Toxicol Environ Health A.

[CIT0006] Coassin M, Ursini F, Bindoli A (1992). Antioxidant effect of manganese. Arch Biochem Biophys.

[CIT0007] Dobson AW, Erikson KM, Aschner M (2004). Manganese neurotoxicity. Ann N Y Acad Sci.

[CIT0008] Goering PL, Klaassen CD (1985). Mechanism of manganese-induced tolerance to cadmium lethality and hepatotoxicity. Biochem Pharmacol.

[CIT0009] Günzler VA, Kremers H, Flohe L (1974). An improved coupled test procedure for glutathione peroxidase (EC 1.11.1.9.) in blood. Z Klin Chem Biochem.

[CIT0010] HaMai D, Bondy SC (2004). Oxidative basis of manganese neurotoxicity. Ann N Y Acad Sci.

[CIT0011] Himeno S, Yanagiya T, Enomoto S, Kondo Y, Imura N (2002). Cellular cadmium uptake mediated by the transport system for manganese. Tohoku J Exp Med.

[CIT0012] Himeno S, Yanagiya T, Fujishiro H (2009). The role of zinc transporters in cadmium and manganese transport in mammalian cells. Biochimie.

[CIT0013] Hussain S, Ali SF (1999). Manganese scavenges superoxide and hydroxyl radicals: an in vitro study in rats. Neuroscience Letters.

[CIT0014] International Agency for Research on Cancer Monographs (1993). Cadmium.

[CIT0015] Kotyzová D, Caisová D, Eybl V (1990). The influence of manganese pre-treatment on cadmium toxicity. Cs. fysiologie.

[CIT0016] Macmillan-Crow LA, Cruthirds DL (2001). Invited review: manganese superoxide dismutase in disease. Free Radic Res.

[CIT0017] Mihara M, Uchiyama M (1978). Determination of malondialdehyde precursor in tissues by thiobarbituric acid test. Analyt Biochem.

[CIT0018] Pillai A, Gupta S (2005). Antioxidant enzyme activity and lipid peroxidation in liver of female rats co-exposed to lead and cadmium: effects of vitamin E and Mn2+. Free Radic Res.

[CIT0019] Santamaria AB, Sulsky SI (2010). Risk assessment of an essential element: manganese. J Toxicol Environ Health A.

[CIT0020] Sedlak J, Lindsay RH (1968). Estimation of total, proteinbound and nonprotein sulfhydryl groups in tissue with Ellman‘s reagent. Analyt Biochem.

[CIT0021] Sziráki I, Rauhala P, Chiueh CC (1995). Novel protective effect of manganese against ferrous citrate-induced lipid peroxidation and nigrostriatal neurodegeneration in vivo. Brain Res.

[CIT0022] Sziráki I, Rauhala P, Koh KK, van Bergen P, Chiueh CC (1999). Implications for atypical antioxidative properties of manganese in iron-induced brain lipid peroxidation and copper-dependent low density lipoprotein conjugation. Neurotoxicology.

[CIT0023] Valachová K, Kogan G, Gemeiner P, Šoltés L (2010). Protective effects of manganese(II) chloride on hyaluronan degradation by oxidative system ascorbate plus cupric chloride. Interdisc Toxicol.

[CIT0024] Valko M, Rhodes CJ, Moncol J, Izakovic M, Mazur M (2006). Free radicals, metals and antioxidants in oxidative stress-induced cancer. Chem Biol Interact.

[CIT0025] Waalkes MP (2000). Cadmium carcinogenesis in review. J Inorg Biochem.

[CIT0026] World Health Organisation (1992). Environmental Health Criteria, Cadmium.

